# A reassessment of the Japanese clinical diagnostic criteria of familial hypercholesterolemia in a hospital-based cohort using comprehensive genetic analysis

**DOI:** 10.1016/j.plabm.2020.e00180

**Published:** 2020-10-19

**Authors:** Hayato Tada, Hirofumi Okada, Akihiro Nomura, Atsushi Nohara, Soichiro Usui, Kenji Sakata, Masayuki Takamura, Masa-aki Kawashiri

**Affiliations:** a Department of Cardiovascular Medicine, Kanazawa University Graduate School of Medical Sciences, Kanazawa, Japan; b Department of Genetics, Ishikawa Prefectural Central Hospital, Kanazawa, Japan

**Keywords:** Achilles tendon thickness, Familial hypercholesterolemia, Proprotein convertase subtilisin/kexin type 9, Low-density lipoprotein receptor

## Abstract

**Background:**

Clinical diagnostic criteria of familial hypercholesterolemia (FH) in Japan include LDL cholesterol ​≥ ​180 ​mg/dL, Achilles tendon thickness ​≥ ​9.0 ​mm, and family history. However, few data exist regarding its validation.

**Design and Methods:**

A series of 680 participants, with a mean LDL cholesterol of 175 ​mg/dL were enrolled at Kanazawa University Hospital between 2006 and 2018. All had full assessments of, LDL cholesterol, Achilles tendon X-rays, family history records, and genetic analysis of FH-associated genes (*LDLR*, *APOB*, and *PCSK9*). The area under the curve (AUC) of receiver operating characteristic (ROC) curve analysis predicting the presence of FH mutations by each clinical marker were assessed.

**Results:**

The optimal cutoff values predicting the presence of an FH-associated mutation were 181 ​mg/dL for LDL cholesterol and ≥7.0 ​mm for Achilles tendon thickness. AUCs predicting FH mutations were 0.827 for Achilles tendon thickness ≥9.0 ​mm, 0.889 for LDL cholesterol ≥180 ​mg/dL, and 0.906 for family history. If Achilles tendon thickness ≥7.0 ​mm was used as a clinical criterion, then 41 participants (6%) were newly diagnosed with FH and 86 (12%) were newly misclassified as FH.

**Conclusions:**

Current clinical diagnostic criteria of FH were validated in this cohort. We recommend considering a tentative diagnosis of “potential FH” if the Achilles tendon thickness is ​≥ ​7.0 ​mm and <9.0 ​mm rather than dismissing a diagnosis of FH.

## Highlights

• Clinical diagnostic criteria of FH in Japan were well validated.
• The best cutoff value of ATT for predicting FH mutation was 7.0 ​mm.
• Threshold of ATT ≥7.0 ​mm increased the sensitivity to diagnose FH.

## 1 Introduction

Familial hypercholesterolemia (FH; OMIM #143890) is characterized by a clinical triad of primary hyper-low-density lipoprotein (LDL)-cholesterolemia, tendon xanthomas, and premature coronary artery disease (CAD) [1]. Even though FH is thought to be caused by deleterious mutations in genes associated with LDL metabolism such as LDL receptor (*LDLR*), apolipoprotein B (*APOB*), and proprotein convertase subtilisin/kexin type 9 (*PCSK9*), and accumulation of LDL-raising single nucleotide polymorphisms [2], genetic testing is rarely used in clinical settings [3]. The criteria for clinical diagnosis of FH vary with world region and including those of the Dutch Lipid Clinical Network (DLCN) [4], the Simon-Bloom diagnostic criteria [5], and Make Early Diagnosis to Prevent Early Death (MEDPED) [6]. The DLCN scoring system is widely used but complicated to calculate, and the definition of tendon xanthomas is not clear. The Japan Atherosclerosis Society criteria include the clinical triad noted above. The criteria are LDL cholesterol ≥180 ​mg/dL; tendon xanthomas on the back of the hands, elbows, or knees, or Achilles tendon hypertrophy; and a family history of FH or second-degree relatives with a history of premature CAD [7]. An Achilles tendon thickness ≥9.0 ​mm or xanthoma tuberosum are confirmed on X-rays. Japanese clinical criteria are easy to use, and the definition of tendon xanthoma is clear. However, the LDL cholesterol value was determined by classical analysis of only *LDLR* gene mutations [8], Achilles tendon thickness was not determined based on a study through genetic analysis in FH patients [9]. Moreover, we frequently experience patients whose LDL cholesterol are extremely elevated (~300 ​mg/dL), thus, they are highly likely to be FH; however, their Achilles tendon thickness is not always larger than ​≥ ​9.0 ​mm, and their family history information is sometimes unclear, leading to dismiss their diagnosis as FH. The study objective was to reassess the current Japanese FH criteria with comprehensive genetic analysis including the *PCSK9* gene.

## 2 Materials and methods

### 2.1 Study population

A group of 1981 patients at Kanazawa University Hospital with a measurement of Achilles tendon thickness for any reason between April 2006 to March 2018 were eligible. Screening excluded 1092 patients for lack of lipid profiles and/or comprehensive genetic analysis, six with homozygous or compound heterozygous FH, one with autosomal recessive hypercholesterolemia, and 202 with a history of lipid-lowering therapy. We did not include patients whose data were used during the process of the current Japanese guideline (XX). A cohort of 680 participants with a mean age of 50 ​± ​18 years was included in this retrospective analysis. There were 344 men (51%), and 145 (21%) had a history of CAD. The baseline data included a medical history review, a physical examination, and a blood testings. Most study participants were inpatients, making it possible to obtain fasting blood samples.

### 2.2 Achilles tendon thickness

Two experienced lipidologists (HT, HO) evaluated the Achilles tendon X-rays. Intra- and inter-observer variability were assessed by the Bland–Altmann method, and the coefficient of variation (CV) was calculated for 40 randomly selected participants.

### 2.3 Genetic analysis

Genomic DNA was isolated from peripheral white blood cells and polymerase chain reaction assays were performed in clinically diagnosed FH participants following standard procedures. The exome regions of 21 dyslipidemia-related genes with Mendelian inheritance, including three FH genes (*LDLR*, *APOB*, and *PCSK9*) were sequenced. The pathogenicity of the variants were determined by allele frequency, *in-silico* analysis, and Clinvar (https://www.clinicalgenome.org/data-sharing/clinvar) as previously described [10].

### 2.4 Ethical considerations

The study was approved by the Ethics Committee of Kanazawa University and conducted following the ethical standards of the responsible committee on human experimentation (institutional and national) and with the Helsinki Declaration of 1975, as revised in 2008. The study participants gave informed consent for genetic analysis before inclusion.

### 2.5 Biochemical analysis

Blood samples were collected after overnight fasting, and serum total cholesterol, triglycerides and high-density lipoprotein (HDL) cholesterol were assayed enzymatically with an autoanalyzer (Qualigent, Sekisui Medical, Tokyo, Japan) as previously described [11]. If triglycerides were <400 ​mg/dL, then LDL cholesterol concentration was calculated with the Friedewald equation; if not, then it was determined enzymatically. The initial data was obtained prior to the introduction of lipid-lowering treatments.

### 2.6 Clinical evaluation

Hypertension was defined as a systolic blood pressure of ≥140 ​mmHg and a diastolic blood pressure of ≥90 ​mmHg or the use of antihypertensive medications. Coexisting diabetes was defined as described by the Japan Diabetes Society or the use of diabetes medications. CAD was defined by the presence of angina pectoris, myocardial infarction, or severe stenosis of the coronary artery or arteries identified either on an angiogram or by computed tomography [12].

### 2.7 Statistical analysis

Categorical variables were reported as percentages and compared with Fisher’s exact test or the chi-square test, whichever was appropriate. Continuous variables with a normal distribution were reported as means ​± ​standard deviation (SD). Variables that were not normally distributed were reported as medians and interquartile range (IQR). Mean values of continuous variables were compared with Student’s *t*-test for independent data; and median values were compared with the nonparametric Wilcoxon Mann–Whitney rank sum test, or the chi-squared test was used for categorical variables with Fisher’s post-hoc test. Multivariate logistic analysis, including factors possibly associated with Achilles tendon thickness or CAD, was used to determine the significance of the association of patient variables and outcomes. Odds ratios (ORs) and their 95% confidence ratios (CIs) were calculated. Receiver operating characteristic (ROC) curve analysis was performed and areas under the curve (AUCs) were calculated to estimate the predictive performance of patient variables. The statistical analysis was conducted with R statistics (https://www.r-project.org). *P*-values of <0.05 were considered statistically significant.

## 3 Results

### 3.1 Participant characteristics

The clinical characteristics of the study participants are shown in Table 1. The mean age was 50 ​± ​18 years, mean LDL cholesterol was 175 ​mg/dL, and mean Achilles tendon thickness was 7.2 ​mm. A heterozygous FH-associated mutation was identified in 175 of the 680 participants (26%), 156 with *LDLR* mutations and 19 with exhibited *PCSK9* mutations (Supplemental Table 1). As shown in Table 1, there were significant differences in the presence of mutations in participants with and without CAD.

**Table 1:** Baseline characteristics of the study participants.

−	All	CAD		
	(n ​= ​680)	YES (n ​= ​145)	NO (n ​= ​535)	*p*-value
Age (years)	50 ​± ​18	61 ​± ​13	47 ​± ​18	<2 ​× ​10^−16^
Male	344 (51%)	104 (72%)	240 (45%)	1.3 ​× ​10^−8^
Body weight (kg)	62 ​± ​14	67 ​± ​14	61 ​± ​14	2.8 ​× ​10^−6^
Hypertension	247 (36%)	115 (79%)	132 (25%)	<2 ​× ​10^−16^
Diabetes	125 (18%)	53 (37%)	72 (13%)	4.2 ​× ​10^−10^
Smoking	242 (36%)	111 (77%)	131 (24%)	<2 ​× ​10^−16^
Total cholesterol (mg/dL)	264 ​± ​78	271 ​± ​74	255 ​± ​79	9.6 ​× ​10^−4^
Triglyceride (mg/dL)	117 [78–182]	110 [74–181]	128 [100–182]	0.08
HDL cholesterol (mg/dL)	53 ​± ​17	45 ​± ​12	55 ​± ​18	<2 ​× ​10^−16^
LDL cholesterol (mg/dL)	175 ​± ​72	179 ​± ​71	168 ​± ​73	3.8 ​× ​10^−4^
Family history	180 (26%)	28 (19%)	152 (28%)	0.04
FH mutation	175 (26%)	27 (19%)	148 (28%)	0.04
Achilles tendon thickness (mm)	7.2 ​± ​3.0	7.9 ​± ​3.6	7.0 ​± ​2.8	0.008

FH, familial hypercholesterolemia; CAD, coronary artery disease.

### 3.2 Precision of achilles tendon thickness measurement

**Fig. 1:**
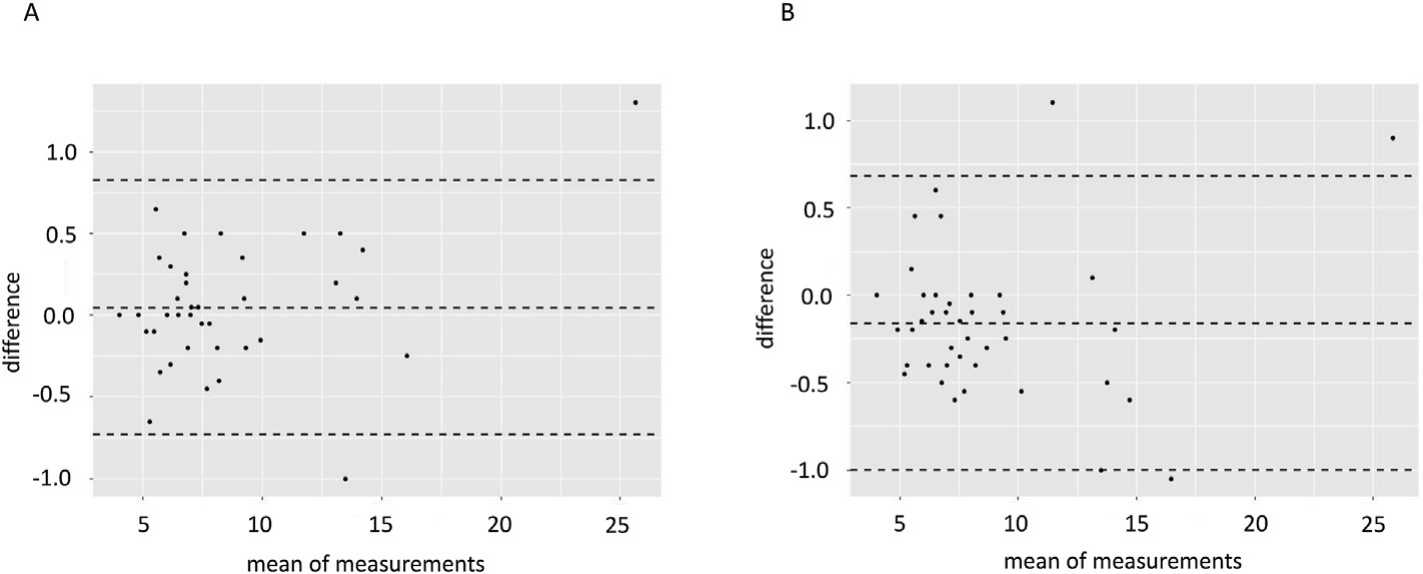
Bland–Altman plots of Achilles tendon thickness measurement found good agreement of (A) intra-observer and (B) inter-observer measurements.

As shown in the Bland–Altman plot in (Fig. 1), most data points were well within the interval defined by the mean ​± ​2 SDs. The CV of the intra-observer measurements was 8.0%, and that of the inter-observer measurements was 11.2%.

### 3.3 Distribution of achilles tendon thickness by FH mutation status

**Fig. 2:**
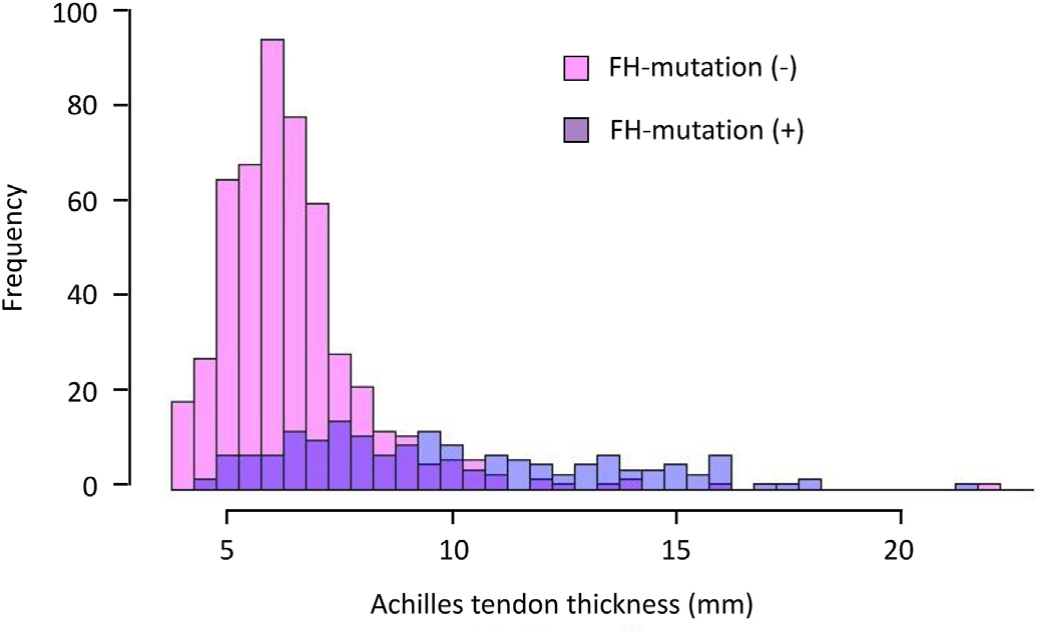
A histogram of Achilles tendon thickness and genetic status of FH shows that most, but not all participants with an Achilles tendon thickness of <9 ​mm lacked an FH mutation (pink) and some participants with a thickness ​≥ ​9 ​mm did carry an FH mutation (purple). (For interpretation of the references to colour in this figure legend, the reader is referred to the Web version of this article.)

As shown in Fig. 2, most participants without an FH mutation an Achilles tendon thickness of <9.0 ​mm. Many participants with an Achilles tendon thickness ≥9.0 ​mm had no FH mutations and many with FH mutations had Achilles tendons <9.0 ​mm thick.

### 3.4 Factors associated with achilles tendon thickness

**Fig. 3:**
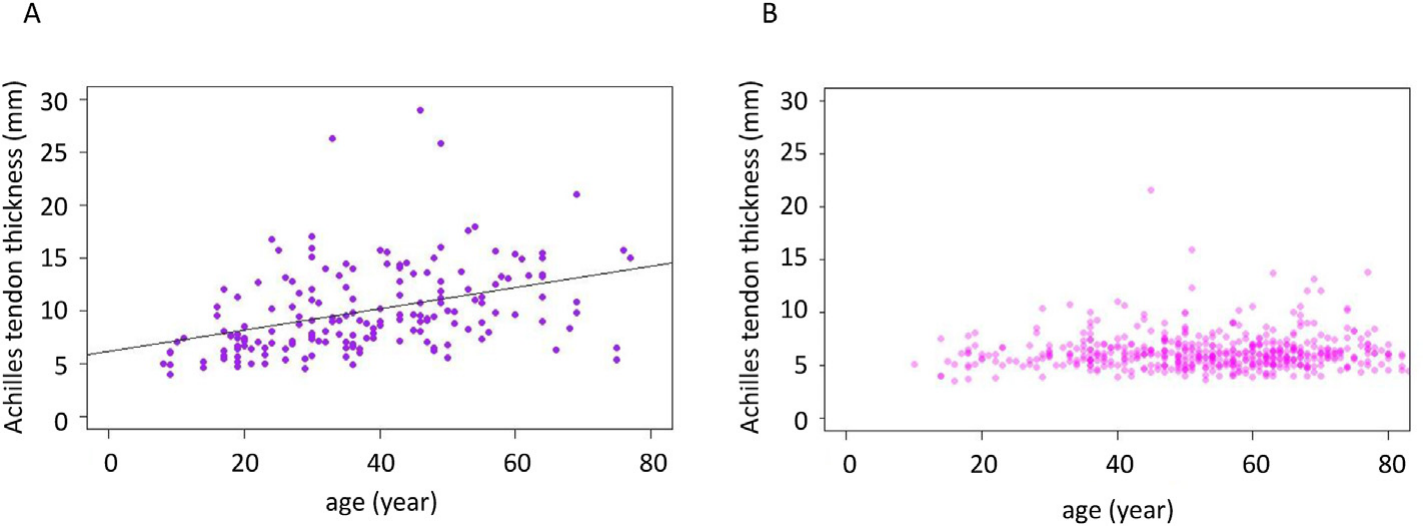
Scatter plots show that a significant correlation of age and Achilles tendon thickness was found in those (A) with an FH mutation (purple dots) *r* ​= ​0.391, *p* ​= ​8.7 ​× ​10^−8^) and (B) not in those not carrying an FH mutation (pink dots). (For interpretation of the references to colour in this figure legend, the reader is referred to the Web version of this article.)

Multivariate logistic regression analysis with an Achilles tendon thickness ≥9.0 ​mm as the reference (Table 2) revealed that age (OR, 1.04; 95% CI: 1.02–1.06, *p* ​= ​4.7 ​× ​10^−6^), body weight (OR, 1.04; 95% CI: 1.02–1.06, *p* ​= ​0.003), diabetes (OR, 2.59; 95% CI: 1.20–5.65, *p* ​= ​0.016), triglycerides (OR, 1.001; 95% CI: 1.000–1.002, *p* ​= ​0.03), LDL cholesterol per 10 ​mg/dL (OR, 1.12; 95% CI: ​= ​1.07–1.17, *p* ​= ​1.4 ​× ​10^−6^), and FH mutation (OR, 25.9; 95% CI: 12.9–55.0, *p* ​< ​2 ​× ​10^−16^) were independently associated with Achilles tendon thickness. A similar trend was seen for body mass index (OR, 1.08; 95% CI: 1.01–1.16, *p* ​= ​0.0256) in a model with body mass index replacing body weight (Supplemental Table 2). The magnitude of the effect of FH mutation was by far the largest. When the analysis was adjusted for variables associated with Achilles tendon thickness, and participants were stratified by the presence or absence of FH mutation, a significant correlation of age and Achilles tendon thickness was found only in those with FH mutation (*r* ​= ​0.391, *p* ​= ​8.7 ​× ​10^−8^, Fig. 3).

**Table 2:** Factors associated with Achilles tendon thickness.

Variable	OR (95% CI)	*p*-value
Age	1.04 (1.02–1.06)	4.7 ​× ​10^−6^
Gender	1.02 (0.52–1.98)	0.95
Body weight	1.04 (1.02–1.06)	0.0003
Hypertension	0.87 (0.42–1.85)	0.72
Diabetes	2.59 (1.20–5.65)	0.016
Smoking	1.66 (0.79–3.54)	0.18
Triglyceride	1.001 (1.000–1.002)	0.03
HDL cholesterol	1.006 (0.98–1.02)	0.50
LDL cholesterol (per 10 ​mg)	1.12 (1.07–1.17)	1.4 ​× ​10^−6^
FH mutation	25.9 (12.9–55.0)	<2 ​× ​10^−16^

FH, familial hypercholesterolemia; OR, odds ratio, CI, confidence interval.

### 3.5 Factors associated with CAD

Multivariate logistic regression analysis (Table 3) revealed that age (OR, 1.06; 95% CI: 1.04–1.08, *p* ​= ​1.4 ​× ​10^−8^), hypertension (OR, 4.21; 95% CI: 2.41–7.47, *p* ​= ​1.4 ​× ​10^−8^), smoking (OR, 5.63, 95% CI: 2.91–11.31, *p* ​= ​5.3 ​× ​10^−7^), triglycerides (OR, 1.002; 95% CI: 1.00–1.003, *p* ​= ​0.0083), HDL cholesterol (OR, 0.96; 95% CI: 0.94–0.98, *p* ​= ​8.0 ​× ​10^−5^), FH mutation (OR, 2.32; 95% CI: 1.07–5.05, *p* ​= ​0.03), and Achilles tendon thickness ​≥ ​9.0 ​mm (OR, 1.99; 95% CI: 1.01–3.02, *p* ​= ​0.04) were independently associated with the presence of CAD. A change in Achilles tendon threshold thickness to ≥7.0 ​mm did not result in a change in the results of the multivariate analysis (Supplemental Table 3).

**Table 3:** Factors associated with coronary artery disease.

Variable	OR (95% CI)	p-value
Age	1.06 (1.04–1.08)	1.4 ​× ​10^−8^
Gender	0.77 (0.38–1.50)	0.451
Body weight	1.01 (0.99–1.03)	0.27
Hypertension	4.21 (2.42–7.47)	5.5 ​× ​10^−7^
Diabetes	0.86 (0.49–1.50)	0.607
Smoking	5.63 (2.91–11.3)	5.3 ​× ​10^−7^
Triglyceride	1.002 (1.000–1.003)	0.008
HDL cholesterol	0.96 (0.94–0.98)	8.0 ​× ​10^−5^
LDL cholesterol	1.007 (0.97–1.05)	0.729
FH mutation	2.32 (1.07–5.05)	0.032
Achilles tendon thickness ​≥ ​9.0 ​mm	1.99 (1.01–3.02)	0.04

FH, familial hypercholesterolemia; OR, odds ratio; CI, confidence interval.

### 3.6 Predictive value of components of the current guideline

The AUCs of each component in the current Japanese guideline, Achilles tendon thickness ≥9.0 ​mm, LDL cholesterol ≥180 ​mg/mL, and family history of FH are shown in Supplemental Fig. 1. ROC curve analysis revealed AUCs of 0.827 (95% CI: 0.794–0.856) for Achilles tendon thickness, 0.889 (95% CI: 0.861–0.918) for LDL cholesterol, and 0.906 (95% CI: 0.886–0.924) for family history of FH. Combining the three components increased the value to 0.975 (95% CI: 0.956–0.994). The cutoff values with highest sensitivity and specificity were 181 ​mg/dL for LDL cholesterol (sensitivity, 0.857; specificity, 0.798; positive predictive value, 0.595; and negative predictive value, 0.942) and ≥7.0 ​mm for Achilles tendon thickness (sensitivity, 0.743; specificity, 0.802; positive predictive value, 0.565; and negative predictive value, 0.900). Sensitivities, specificities, positive predictive values, and negative predictive values are illustrated in Table 4.

**Table 4:** Sensitivities, specificities, positive predictive values, and negative predictive values.

Component	Sensitivity (%)	Specificity (%)	PPV (%)	NPV (%)
Achilles tendon thickness ​≥ ​7.0 ​mm	74.3	80.2	56.5	90.4
Achilles tendon thickness ​≥ ​9.0 ​mm	53.1	93.5	73.8	85.2
Family history	99.4	98.9	96.7	99.8
LDL cholesterol ​≥ ​180 ​mg/dL	88.0	74.9	54.8	94.7

PPV, positive predictive value; NPV, negative predictive value.

### 3.7 Reclassification of FH by achilles tendon thickness

ROC curve analysis found that prediction of FH mutation status was significantly improved from 0.733 (95% CI: 0.694–0.772) to 0.765 (95% CI: 0.723–0.802, *p* ​= ​0.042, Supplemental Fig. 2). Forty-one participants (6%) with FH mutation whose had an Achilles tendon thickness between 7.0 and 9.0 ​mm (Table 5) and were not diagnosed with FH by the current threshold of 9.0 ​mm. Eighty-six participants (12%) with an Achilles tendon thickness of 7.0–9.0 ​mm did not have FH mutations (Table 5) and would potentially have been misdiagnosed.

**Table 5:** Reclassification of threshold Achilles tendon thickness.

	ATT <7.0 ​mm	7.0 ​mm ​≤ ​ATT <9.0 ​mm	9.0 ​mm ​≤ ​ATT
FH gene (+)	41	41	93
FH gene (−)	386	86	33

ATT, Achilles tendon thickness; FH, familial hypercholesterolemia.

## 4 Discussion

The findings of comprehensive genetic analysis including the *LDLR*, *APOB* and *PCSK9* genes were used to reassess the current Japanese clinical diagnostic criteria of FH. All three components, LDL cholesterol ≥180 ​mg/dL, Achilles tendon thickness ≥9.0 ​mm, and family history of FH were well supported. The best cutoff values for predicting FH mutation were 181 ​mg/dL for LDL cholesterol and 7.0 ​mm for Achilles tendon thickness. Changing the threshold of Achilles tendon thickness from 9.0 to 7.0 ​mm increased the sensitivity to diagnose patients with genetic mutation, but the specificity decreased. FH is a common genetic cause of premature CAD because of lifelong elevated plasma LDL cholesterol. Recent findings from Japan, the USA, and Europe estimate FH prevalence as high as 1 in 200 in people in general population [[13], [14], [15], [16]]. Early diagnosis and prompt LDL-lowering treatment improve the prognosis [17,18], but FH is vastly underdiagnosed worldwide for some European nations [19]. The current clinical diagnosis of FH depends on scoring systems like the DLCN [4] and those that depend on phenotype including hypercholesterolemia, tendon xanthomas, and family history of FH and/or premature CAD, like the Simon Broome Diagnostic Criteria [5], and the Japanese diagnostic criteria [7]. Other systems like the MEDPED diagnostic criteria focus only on cholesterol and family history [6]. The systems are hard to compare, but a diagnosis of FH leads to improved risk discrimination, cascade screening, and possible early diagnosis of FH of relatives. The study findings validated the current clinical diagnostic criteria of FH in Japan and indicate that a lower threshold Achilles tendon thickness might be considered. Achilles tendon thickness had by far the closest association with FH mutation status, but other clinical characteristics, including age, body weight or body mass index, diabetes, triglycerides, and LDL cholesterol were found to be associated with Achilles tendon thickness. As others have also reported that age was independently associated with Achilles tendon thickness, additional clinical characteristics may need to be considered if the thickness is borderline [20]. Based on this notion, careful attention should be paid when assessing the Achilles tendon thickness among young individuals.

The question of what underlies FH, whether it is a monogenic disorder associated with LDL catabolism, an inherited disorder associated with elevated LDL, or hypercholesterolemia with xanthomas remains unanswered. Regardless, preventing the development of CAD by early diagnosis of FH should be the clinical objective. We recommend considering a tentative diagnosis of “potential FH” if the Achilles tendon thickness is ​≥ ​7.0 ​mm and <9.0 ​mm rather than dismissing a diagnosis of FH.

## 5 Study limitations

This study’s limitations include its retrospective, cross-sectional, observational design. However, it included a large sample of the Japanese population, which adds to our understanding of FH across ethnicities. In addition, the findings of factors associated with CAD were consistent with previous reports. Second, including participants whose Achilles tendon thickness had been measured for any reason could have introduced selection bias. Third, genetic analysis was not performed in all participants with Achilles tendon thickness measurements. Fourth, the small proportion of participants younger than 15 years of age limited the power test the association of age and Achilles tendon thickness. Fifth, we assessed how the clinical components were associated with the presence of FH-mutation in this study. On the other hand, around 30% of “clinical FH” do not exhibit FH-mutation, suggesting that the current genetic analysis may not be perfect. It may cause some bias and/or misunderstandings. However, our genetic assessments could be one of the most comprehensive ones in the world.

## 6 Conclusions

The current clinical diagnostic criteria of FH were validated by comprehensive genetic analysis including *PCSK9* gene. We recommend considering a tentative diagnosis of “potential FH” if the Achilles tendon thickness is ​≥ ​7.0 ​mm and <9.0 ​mm rather than dismissing a diagnosis of FH.

## Contributions

Hayato Tada, Hirofumi Okada, Akihiro Nomura, Atsushi Nohara, Masayuki Takamura, and Masa-aki Kawashiri were involved in the recruitment of patients. Hayato Tada, and Masa-aki Kawashiri were involved in the conception and design of the study/analyses. Hayato Tada, Hirofumi Okada, and Akihiro Nomura obtained the data and performed statistical analyses. Hayato Tada, Hirofumi Okada, Akihiro Nomura, Atsushi Nohara, Masayuki Takamura, and Masa-aki Kawashiri were involved in data interpretation and manuscript drafting and reviewing. All authors have approved the final version of the article.

## Sources of funding

This work was supported by scientific research grants from the 10.13039/501100001700Ministry of Education, Science, and Culture of Japan (No. 16K19394, and 18K08064), Health, Labour and Welfare Sciences Research Grant for Research on Rare and Intractable Diseases, 10.13039/501100007263Astellas Foundation for Research on Metabolic Disorders, and ONO Medical Research Foundation.

## Role of the funder/sponsor

The funding sources had no role in the design and conduct of this study; collection, management, analysis, and interpretation of the data; preparation, review, or approval of the manuscript; and decision to submit the manuscript for publication.

## Declaration of competing interest

None.

## References

[1] Mabuchi H. (2017). Half a century tales of familial hypercholesterolemia (FH) in Japan. J. Atherosclerosis Thromb..

[2] Talmud P.J., Shah S., Whittall R., Futema M., Howard P., Cooper J.A. (2013). Use of low-density lipoprotein cholesterol gene score to distinguish patients with polygenic and monogenic familial hypercholesterolaemia: a case-control study. Lancet.

[3] Sturm A.C., Knowles J.W., Gidding S.S., Ahmad Z.S., Ahmed C.D., Ballantyne C.M. (2018). Clinical genetic testing for familial hypercholesterolemia: JACC scientific expert panel. J. Am. Coll. Cardiol..

[4] Austin M.A., Hutter C.M., Zimmern R.L., Humphries S.E. (2004). Genetic causes of monogenic heterozygous familial hypercholesterolemia: a HuGE prevalence review. Am. J. Epidemiol..

[5] Scientific Steering Committee on behalf of the Simon Broome Register Group (1991). Risk of fatal coronary heart disease in familial hypercholesterolaemia. BMJ.

[6] Williams R.R., Hunt S.C., Schumacher M.C., Hegele R.A., Leppert M.F., Ludwig E.H. (1993). Diagnosing heterozygous familial hypercholesterolemia using new practical criteria validated by molecular genetics. Am. J. Cardiol..

[7] Harada-Shiba M., Arai H., Ishigaki Y., Ishibashi S., Okamura T., Ogura M. (2018). Guidelines for diagnosis and treatment of familial hypercholesterolemia 2017. J. Atherosclerosis Thromb..

[8] Harada-Shiba M., Arai H., Okamura T., Yokote K., Oikawa S., Nohara A. (2012). Multicenter study to determine the diagnosis criteria of heterozygous familial hypercholesterolemia in Japan. J. Atherosclerosis Thromb..

[9] Mabuchi H., Ito S., Haba T., Ueda K., Ueda R. (1977). Discrimination of familial hypercholesterolemia and secondary hypercholesterolemia by Achilles’ tendon thickness. Atherosclerosis.

[10] Tada H., Kawashiri M.A., Nomura A., Teramoto R., Hosomichi K., Nohara A. (2018). Oligogenic familial hypercholesterolemia, LDL cholesterol, and coronary artery disease. J. Clin. Lipidol..

[11] Tada H., Kawashiri M.A., Takata M., Matsunami K., Imamura A., Matsuyama M. (2015). Infantile cases of sitosterolemia with novel mutations in the ABCG5 gene: extreme hypercholesterolemia is exacerbated by breastfeeding. JIMD Rep..

[12] Tada H., Kawashiri M.A., Yoshida T., Teramoto R., Nohara A., Konno T. (2016). Lipoprotein(a) in familial hypercholesterolemia with proprotein convertase subtilisin/kexin type 9 (PCSK9) gain-of-function mutations. Circ. J..

[13] Mabuchi H., Nohara A., Noguchi T., Kobayashi J., Kawashiri M.A., Tada H. (2011). Molecular genetic epidemiology of homozygous familial hypercholesterolemia in the Hokuriku district of Japan. Atherosclerosis.

[14] Benn M., Watts G.F., Tybjærg-Hansen A., Nordestgaard B.G. (2016). Mutations causative of familial hypercholesterolaemia: screening of 98 098 individuals from the Copenhagen General Population Study estimated a prevalence of 1 in 217. Eur. Heart J..

[15] de Ferranti S.D., Rodday A.M., Mendelson M.M., Wong J.B., Leslie L.K., Sheldrick R.C. (2016). Prevalence of familial hypercholesterolemia in the 1999 to 2012 United States national Health and nutrition examination surveys (NHANES). Circulation.

[16] Wald D.S., Bestwick J.P., Morris J.K., Whyte K., Jenkins L., Wald N.J. (2016). Child-parent familial hypercholesterolemia screening in primary care. N. Engl. J. Med..

[17] Avis H.J., Vissers M.N., Stein E.A., Wijburg F.A., Trip M.D., Kastelein J.J. (2007). A systematic reviewand meta-analysis of statin therapy in children with familial hypercholesterolemia. Arterioscler. Thromb. Vasc. Biol..

[18] Arambepola C., Farmer A.J., Perera R., Neil H.A. (2007). Statin treatment for children and adolescents with heterozygous familial hypercholesterolaemia: a systematic review and meta-analysis. Atherosclerosis.

[19] Nordestgaard B.G., Chapman M.J., Humphries S.E., Ginsberg H.N., Masana L., Descamps O.S. (2013). Familial hypercholesterolaemia is underdiagnosed and undertreated in the general population: guidance for clinicians to prevent coronary heart disease: consensus statement of the European Atherosclerosis Society. Eur. Heart J..

[20] Michikura M., Ogura M., Yamamoto M., Sekimoto M., Fuke C., Hori M. (2017). Achilles tendon ultrasonography for diagnosis of familial hypercholesterolemia among Japanese subjects. Circ. J..

