# Supplementary material for: A reassessment of the Japanese clinical diagnostic criteria of familial hypercholesterolemia in a hospital-based cohort using comprehensive genetic analysis
Source: Pract Lab Med. 2020 Oct 19;22:e00180. doi: 10.1016/j.plabm.2020.e00180 (PMC7585136; doi:10.1016/j.plabm.2020.e00180)
Supplement: Multimedia component 1 [file mmc1.docx]

**Supplemental Material**

**A reassessment of the Japanese clinical diagnostic criteria of familial hypercholesterolemia in a hospital-based cohort using comprehensive genetic analysis**

**Supplemental Table 1. Analysis of genomic DNA isolated from peripheral white blood cells of clinically diagnosed FH participants**  1

**Supplemental Table 2. Factors associated with Achilles tendon thickness** 4

**Supplemental Table 3. Factors associated with coronary artery disease** 5

**Supplemental Figure 1. Receiver operating characteristic (ROC) curve analysis for prediction of FH mutation by different components** 6

**Supplemental Figure 2. Receiver-operating characteristic curve analysis for prediction of FH-mutation by Achilles tendon thickness ≥ 9.0 mm or ≥ 7.0 mm**  7

**Supplemental Table 1. Analysis of genomic DNA isolated from peripheral white blood cells of clinically diagnosed FH participants**

| Gene | Nucleotide  Change | Mutation Type | Effect on  Protein | Number of  Patients | Chr:Position (GRCh38) | Exon |
| --- | --- | --- | --- | --- | --- | --- |
| *LDLR* | c.137G>A | Missense | Cys-Tyr | 1 | Chr19:11100292 | 2 |
| *LDLR* | c.344G>A | Missense | Arg-His | 6 | Chr19:11105250 | 4 |
| *LDLR* | c.413C>G | Nonsense | Ser-stop | 2 | Chr19:11105319 | 4 |
| *LDLR* | c.418G>A | Missense | Glu-Lys | 1 | Chr19:11105324 | 4 |
| *LDLR* | c.463T>C | Missense | Cys-Arg | 1 | Chr19:11105369 | 4 |
| *LDLR* | c.478T>C | Missense | Cys-Arg | 1 | Chr19:11105384 | 4 |
| *LDLR* | c.495G>A | Nonsense | Trp-stop | 1 | Chr19:11105401 | 4 |
| *LDLR* | c.550T>C | Missense | Cys-Arg | 1 | Chr19:11105456 | 4 |
| *LDLR* | c.551G>A | Missense | Cys-Tyr | 1 | Chr19:11105457 | 4 |
| *LDLR* | c.611G>C | Missense | Cys-Ser | 4 | Chr19:11105517 | 4 |
| *LDLR* | c.662_665dupACTG | Duplication | Frameshift/stop | 1 | Chr19:11105568 | 4 |
| *LDLR* | c.726G>C | Missense | Gln-His | 1 | Chr19:11106596 | 5 |
| *LDLR* | c.1012T>A | Missense | Cys-Ser | 6 | Chr19:11110723 | 7 |
| *LDLR* | c.1048C>T | Nonsense | Arg-stop | 2 | Chr19:11110759 | 7 |
| *LDLR* | c.1060G>A | Missense | Asp-Asn | 1 | Chr19:11110771 | 7 |
| *LDLR* | c.1062dupT | Duplication | Frameshift/stop | 7 | Chr19:11222191 | 8 |
| *LDLR* | c.1297G>C | Missense | Asp-His | 10 | Chr19:11113388 | 9 |
| *LDLR* | c.1325A>G | Missense | Tyr-Cys | 1 | Chr19:11113416 | 9 |
| *LDLR* | c.1432G>A | Missense | Gly-Ala | 6 | Chr19:11113608 | 10 |
| *LDLR* | c.1474G>A | Missense | Asp-Asn | 8 | Chr19:11113650 | 10 |
| *LDLR* | c.1567G>A | Missense | Val-Met | 10 | Chr19:11113743 | 10 |
| *LDLR* | c.1652_1662del | Deletion | Frameshift/stop | 1 | Chr19:11116159 | 11 |
| *LDLR* | c.1689dupC | Duplication | Frameshift/stop | 8 | Chr19:11116196 | 11 |
| *LDLR* | c.1702C>G | Missense | Leu-Val | 1 | Chr19:11116209 | 11 |
| *LDLR* | c.1731G>T | Missense | Trp-Cys | 1 | Chr19:11116884 | 12 |
| *LDLR* | c.1778dupG | Duplication | Frameshift/stop | 2 | Chr19:11116931 | 12 |
| *LDLR* | c.1871_1873delTCA | Deletion | Frameshift/stop | 1 | Chr19:11120117 | 13 |
| *LDLR* | c.1871_1873delTCA | Deletion | 603 Ile deletion | 1 | Chr19:11120117 | 13 |
| *LDLR* | c.1931delA | Deletion | Frameshift/stop | 1 | Chr19:11120177 | 13 |
| *LDLR* | c.2026G>A | Missense | Gly-Ser | 1 | Chr19:11120408 | 14 |
| *LDLR* | c.2055delG | Deletion | Frameshift/stop | 1 | Chr19:11120437 | 14 |
| *LDLR* | c.2054C>T | Missense | Pro-Leu | 2 | Chr19:11120436 | 14 |
| *LDLR* | c.2257C>T | Missense | Pro-Ser | 1 | Chr19:11123290 | 15 |
| *LDLR* | c.2431A>T | Nonsense | Lys-stop | 54 | Chr19:11129554 | 17 |
| *LDLR* | c.2141-?_2311+?del | Large deletion | Truncated protein | 3 | NA | 15–18 |
| *LDLR* | c.2312-3C>A | Splicing | exon16 skip | 6 | Chr19:11128005 | IVS15 |
| *PCSK9* | c.94G>A | Missense | Glu-Lys | 19 | Chr1:55039931 | 1 |

LDLR, LDL receptor; PCSK9, proprotein convertase subtilisin/kexin type 9; NA, not available

**Supplemental Table 2. Factors associated with Achilles tendon thickness**

| Variable | OR (95% CI) | *p*-value |
| --- | --- | --- |
| Age | 1.04 (1.02–1.06) | 4.2 × 10^−5^ |
| Gender | 1.48 (0.80–2.76) | 0.214 |
| Body mass index | 1.08 (1.01–1.16) | 0.0256 |
| Hypertension | 0.92 (0.44–1.94) | 0.82 |
| Diabetes | 2.56 (1.19–5.54) | 0.016 |
| Smoking | 1.82 (0.88–3.83) | 0.11 |
| Triglyceride | 1.001 (1.000–1.002) | 0.056 |
| HDL cholesterol | 1.006 (0.98–1.02) | 0.64 |
| LDL cholesterol (per 10 mg) | 1.11 (1.07–1.16) | 1.8 × 10^−6^ |
| FH-mutation | 23.9 (12.0–50.4) | <2 × 10^−16^ |

FH: familial hypercholesterolemia, OR: odds ratio, CI: confidence interval

**Supplemental Table 3. Factors associated with coronary artery disease**

| Variable | OR (95% CI) | p-value |
| --- | --- | --- |
| Age | 1.04 (1.01–1.07) | 5.6 × 10^−6^ |
| Gender | 0.75 (0.34–1.55) | 0.55 |
| Body weight | 1.01 (0.99–1.04) | 0.33 |
| Hypertension | 3.88 (2.02–8.07) | 2.4 × 10^−5^ |
| Diabetes | 0.92 (0.51–1.66) | 0.71 |
| Smoking | 5.02 (2.56–11.9) | 7.7 × 10^−6^ |
| Triglyceride | 1.003 (1.001–1.005) | 0.009 |
| HDL cholesterol | 0.96 (0.94–0.98) | 7.7 × 10^−5^ |
| LDL cholesterol | 1.007 (0.97–1.06) | 0.88 |
| FH-mutation | 2.14 (1.06–4.87) | 0.039 |
| Achilles tendon thickness ≥ 7.0 mm | 2.12 (1.05–3.34) | 0.028 |

FH: familial hypercholesterolemia, OR: odds ratio, CI: confidence interval

**Supplemental Figure 1. Receiver operating characteristic (ROC) curve analysis for prediction of FH mutation by different components**


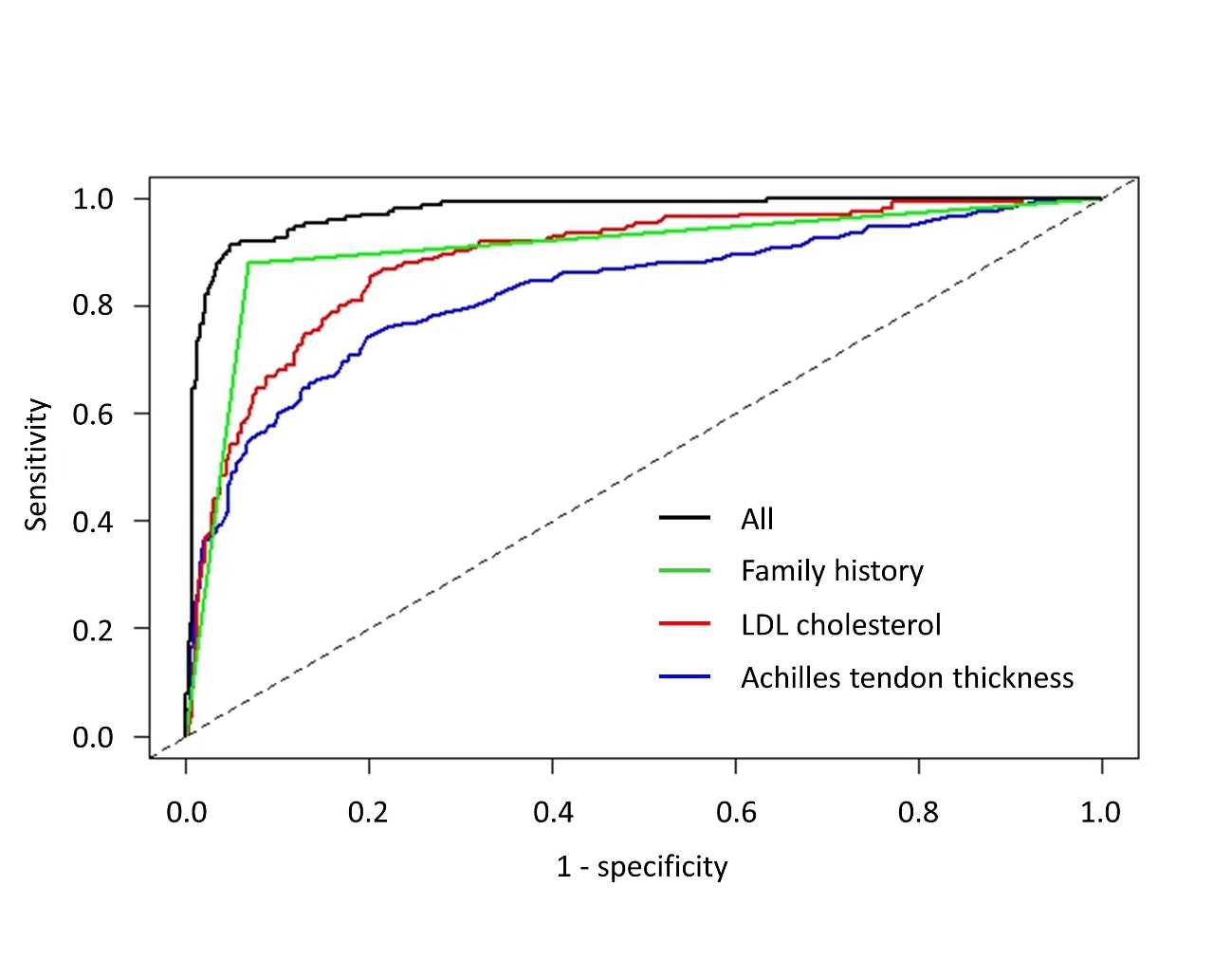


**Supplemental Figure 2
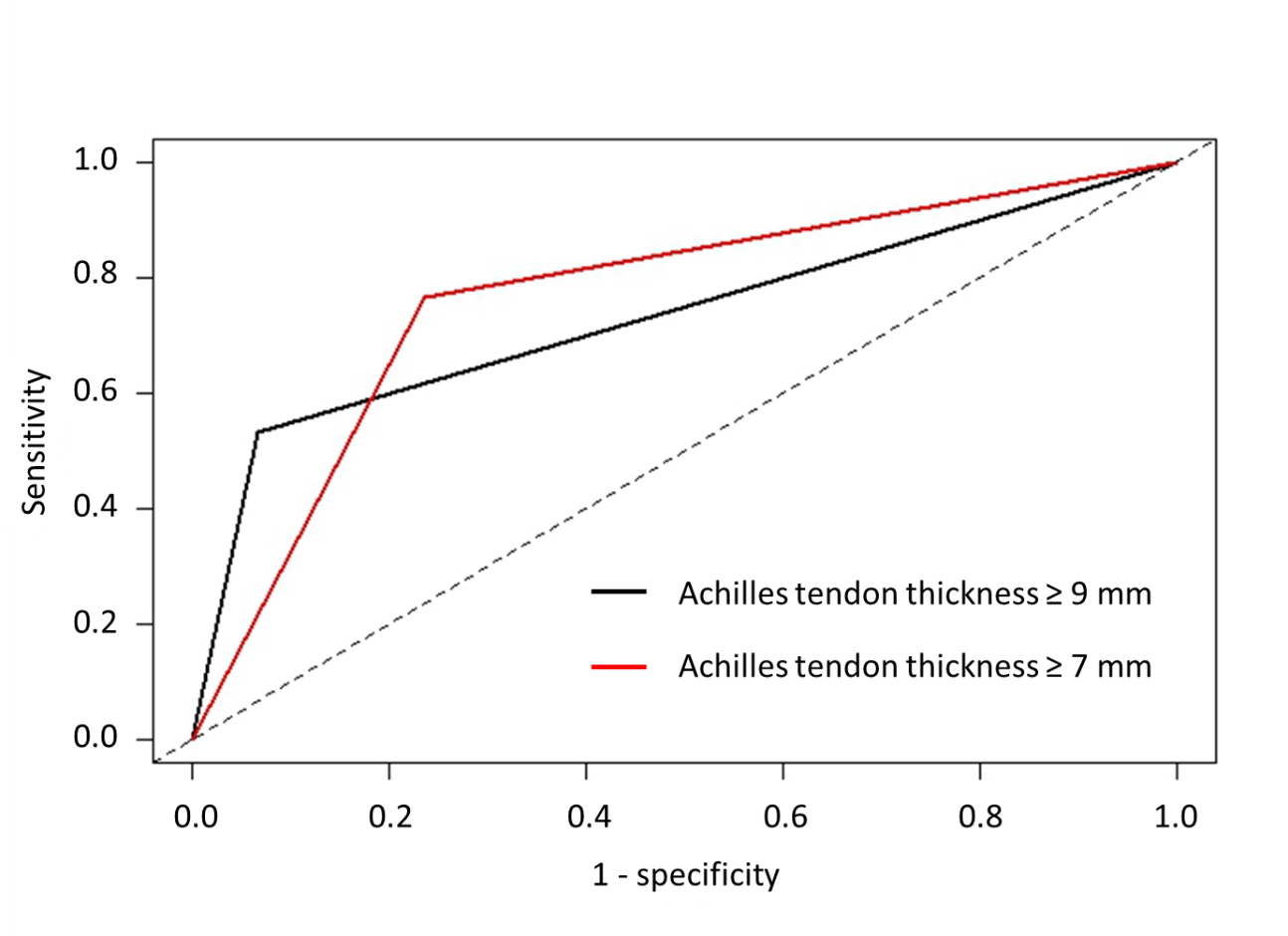
. Receiver-operating characteristic curve analysis for prediction of FH-mutation by Achilles tendon thickness ≥ 9.0 mm or ≥ 7.0 mm**
